# Optogenetics as a neuromodulation tool in cognitive neuroscience

**DOI:** 10.3389/fpsyg.2013.00610

**Published:** 2013-09-06

**Authors:** E. A. Claudia Pama, Lorenza S. Colzato, Bernhard Hommel

**Affiliations:** Cognitive Psychology Unit, Psychology, Leiden Institute for Brain and Cognition, Leiden UniversityLeiden, Netherlands

**Keywords:** optogenetics, neuromodulation, cognitive neuroscience, dopamine, opto-fMRI

Optogenetics may be the answer to a search for temporal and spatial specificity in neuroscience. The well-known trade-off between temporal and spatial specificity might be resolved with this “combination of genetic and optical methods to achieve gain or loss of function of well-defined events in specific cells of living tissue” (Deisseroth, 2011). It is a technology that enables researchers to stimulate cells with light, thereby allowing for the direct control of behavior. Until now, this technique has been applied in animal research only but, as we argue, it holds promise for research in humans as well.

The idea of using light to control cells is not a recent one. Already in 1979, Francis Crick anticipated the struggle of neuroscience to target individual cells *in vivo* without affecting others, and he suggested light as a tool to achieve that. Around that time it became clear that certain microorganisms possess proteins that respond to light. Oesterhelt and Stoeckenius (1971) discovered bacteriorhodopsin, an ion-pump that can be activated by light photons. Other members of this family were identified soon after, including halorhodopsin (Matsuno-Yagi and Mukohata, 1977) and channelrhodopsin (Nagel et al., 2002). In 2005, researchers at Karl Deisseroth's laboratory first demonstrated a single-component optogenetic system (Boyden et al., 2005), and in 2006 the term “optogenetics” was born.

It is beyond the scope of this article to provide an exhaustive discussion of all discoveries that led to what optogenetics is today, or of all the impressive and sophisticated advances to improve the technique. Instead, we will restrict ourselves to briefly introducing the general concept of optogenetics and discussing its potential for cognitive neuroscience.

## The process of optogenetics

**Figure 1:**
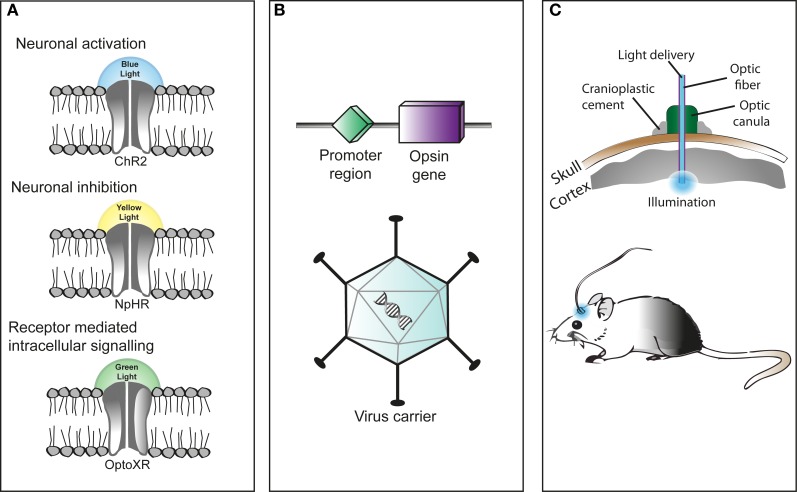
**Optogenetic stimulation consists of several steps. (A)** First, light-responsive proteins called opsins need to be specified. There are two distinct superfamilies: microbical opsins (type I) and animal opsins (type II). Both types require retinal (a vitamin A-related organic cofactor) to convert light into energy, and the binding of retinal renders these opsins rhodopsins (Fenno et al., 2011). Several types of microbial opsins have been identified as suitable for optogenetic control and each type reacts differently to light stimulation of particular wavelengths; e.g., blue light makes channelrhodopsin-2 (ChR2) rapidly depolarize a neuron. The opsins used for optogenetics have different ion conductance regulation properties, acting within timeframes ranging from milliseconds to tens of minutes (Fenno et al., 2011). Examples include ChETAs (modified ChR2 opsins) that allow for ultrafast optogenetic control, and step-function opsins, purposely engineered to show prolonged activity after termination of the light stimulus (Fenno et al., 2011). As for neuronal inhibition, certain light-activated pumps may be used, such as the Natronomonas pharaonis Halorhodopsin (NpHR) ion pump. Here, halorhodopsins hyperpolarize the neuron in reaction to yellow light. Finally, biochemical control can be achieved by using type II animal opsins. Controlling G protein-coupled receptors has now been made possible by modifying them into so-called optoXRs (Airan et al., 2009). These proteins allow for receptor-mediated intracellular signaling by responding to green (500 nm) light (Fenno et al., 2011). **(B)** The next step is to make sure that mammalian cells express microbial opsins. Because simply administering a protein will not work, a gene that encodes for the opsin needs to be introduced to specific cells instead. One possibility is to inject a (harmless) virus to carry the opsin gene into the brain of a mammal. The major drawback of viral expression systems is that they cannot carry large amounts of genetic material. However, the advantage is that opsins are expressed in high levels. Another way to introduce opsins is to use transgenic (knock-in) animals that possess the opsins from birth. This obviously has the advantage of studying the development of a system. However, transgenic animals show lower opsin expression levels. Other options include the use of Cre-driver animals, Cre-dependent viruses or *in utero* electroporation. It is also possible to target circuits by “projection targeting,” where light is delivered to an axon instead of the soma, or to use viruses that transduce along axon terminals. Combination strategies are also possible, as the detailed overview of Fenno et al. (2011) shows. **(C)** Light can be delivered straight into the brain through an optical fiber, using a chronically implanted cannula that is affixed to the skull.

Strictly speaking, optogenetics involves “experimenting with a combination of genetic manipulation and optics” (Kasparov, 2011). It can be used in several animal models, including the *C. elegans*, fly, zebrafish, mouse, rat, and primate (Fenno et al., 2011). Ultimately, optogenetics may be used to control the behavior of freely moving mammals by administering light. However, several steps need to be taken to achieve this, as illustrated in Figure 1.

First, the opsins of interest have to be specified. Optogenetics has mostly focussed on microbial opsins, because these are capable of directly coupling light to rapid ion transport (Yizhar et al., 2011a; Han, 2012). However, since microbial opsins serve strictly as ion flow modulators, animal opsins are being used for biochemical control (Fenno et al., 2011). Neuronal activation can be obtained by illuminating channelrhodopsin-2 (ChR2) with blue light, while neuronal inhibition results from illuminating halorhodopsin (NpHR) with yellow light. Receptor-mediated intracellular signaling can be achieved by shining green light on OptoXRs, see Figure 1A.

The next step is to make sure that mammalian cells express microbial opsins, for example by using virus carrier systems (Figure 1B). The virus carries the opsin gene and is injected into the cell of interest. There are several types of viral vectors that can be used for this purpose, the most common being the Lenti and adeno-associated virus (AAV).

Finally, light has to be delivered into the brain. This can be achieved by using a chronically implanted cannula (affixed to the skull) to which an optical fiber can be attached (Figure 1C). Laser light can then be delivered via this optical fiber directly into the brain (Zhang et al., 2010).

## The application of optogenetics

Many questions in cognitive neuroscience regarding the molecular, cellular and circuit-level underpinnings of behavior still remain unresolved due to the trade-off between temporal and spatial specificity. Since optogenetic tools have become available to the scientific community, numerous studies have applied this method successfully to answer such questions. The advantage of optogenetics over other neuromodulation techniques is its high-temporal specificity combined with cellular precision. For example, although electrical manipulation has a high temporal resolution, it is unable to achieve true inactivation or excitation of individual neurons. Pharmacological and genetic manipulations show the opposite pattern, they can target at least certain kinds or families of neurons but are lacking temporal precision (Fenno et al., 2011). Optogenetics can be applied to very diverse research topics, even outside neuroscience. Here, we will limit ourselves to a few studies that successfully applied optogenetic tools in areas that are relevant for cognitive neuroscience; for a broader picture see the special issue of *Biological Psychiatry* on this topic (Deisseroth, 2012).

Optogenetics has been successfully applied in neuromodulation research. For example, Witten et al. (2011) used optogenetic tools to clarify the relationship between dopamine (DA) neuron firing and positive reinforcement in genetically modified rats. They observed that optical stimulation of DA neurons in the ventral tegmental area of these rats led to vigorous intracranial self-stimulation. Likewise, Tsai et al. (2009) demonstrated that phasic dopaminergic activity is sufficient to mediate mammalian behavioral conditioning, by using an optogenetic approach. They emphasize that integrating optogenetics with other approaches (e.g., electrophysiological, behavioral and electrochemical methods) will reveal relevant interactions of DA neurons with other neuromodulatory circuits (e.g., monoaminergic and opioid circuits). The use of optogenetics further revealed opposite roles of D1+ and D2+ neurons (in the nucleus accumbens) in processing cocaine reward (Lobo et al., 2010). In this study the firing rate of D1+ and D2+ neurons was selectively controlled to investigate the resulting effects on cocaine reward. It was found that activation of D2+ neurons suppresses cocaine reward, while activation of D1+ neurons shows the opposite pattern. Another example of optogenetic neuromodulation shows how symptoms of Parkinson's disease can be either aggravated or improved (Kravitz et al., 2010). Kravitz et al. (2010) modulated the firing activity of single neurons, manipulating either direct or indirect pathways in the basal ganglia. Another study (Bass et al., 2010) showed how neuronal dopamine release patterns could be evoked in the dorsal part of the striatum in living rats. Results like these show that the use of optogenetics can lead to a better understanding of cause-effect relationships, for example in dopamine-based disorders.

The neural underpinnings of sleep have also been investigated with optogenetic methods (Adamantidis et al., 2007; de Lecea et al., 2012). In a recent review de Lecea et al. (2012) discuss the use of optogenetics in sleep research as well as in studying the interactions between neuromodulatory systems [e.g., hypocretin (Hcrt) and locus coeruleus/norepinephrine systems]. The authors stress the importance of optogenetics for controlling neural circuits to examine boundaries between sleep and wakefulness (de Lecea et al., 2012). In line with this, researchers stimulated Hcrt producing neurons in freely moving mice (Adamantidis et al., 2007), which led to an increased probability of sleeping mice becoming awake (either from slow wave sleep or rapid eye movement sleep). Interestingly, Hcrt deficiency is associated with the neurological disorder narcolepsy in which sleeping patterns are altered (Adamantidis et al., 2007), and an optogenetic approach may provide further insights into such disorders.

Another area that has successfully been studied with optogenetics is depression (Lobo et al., 2012). For instance, optogenetic stimulation of the medial prefrontal cortex (mPFC) was found to initiate rapid antidepressant-like responses in mice (Covington et al., 2010). Lobo et al. (2012) stress the importance of using optogenetics to study depression, since it allows for answering important questions regarding depression, which have been left unanswered thus far.

A recent review (Yizhar, 2012) on neural circuitries in social functioning revealed how optogenetics might improve our understanding of social behavior and psychiatric impairments, and possibly lead to the development of novel treatment methods. Yizhar et al. (2011b) propose the excitation and inhibition (E/I) balance hypothesis, stating that imbalance in the inhibition and excitation pattern within neural circuitries is involved in several psychiatric diseases and behavioral deficits (e.g., autism, schizophrenia). Using an optogenetic approach, it was indeed found that elevations of cellular E/I balance in the mPFC led to increased high-frequency power (30–80 Hz range) and behavioral impairment (Yizhar et al., 2011b). Optogenetics has further been applied to cortical oscillations (synchronized neural activity), that are associated with various cognitive processes as well as psychiatric conditions such as anxiety, autism, and schizophrenia (Sohal, 2012). Sohal (2012) demonstrated how a particular class of inhibitory interneurons play a causal role in the occurrence of gamma oscillations, which is important for the way neurons communicate.

In addition, several studies used optogenetics to investigate neural circuits that underlie fear conditioning and memory formation (see Johansen et al., 2012, for a review). Other work on contextual fear memories revealed that optogenetic inhibition of CA1 hippocampal neurons can reverse contextual fear memory recall, even weeks after training (Goshen et al., 2011). Studies like these play an important role in the understanding of fundamental cognitive processes like memory formation, but also in anxiety disorders and posttraumatic stress disorder, which are characterized by disturbing, recurring contextual memories.

Finally, a promising methodological approach of optogenetics is the combination with functional Magnetic Resonance Imaging (fMRI). This combination has been termed “opto-fMRI” or “ofMRI” (Desai et al., 2011; Deisseroth, 2012), and has been applied to several domains (Desai et al., 2011; Abe et al., 2012). Although still facing some challenges (Christie et al., 2012), the combination of fMRI with optogenetics provides a unique possibility to observe how functional changes in the brain are brought about as a result of optogenetic manipulation.

## Conclusion

In sum, optogenetics is a promising tool for cognitive neuroscience and we believe that it might be applied to human subjects in the long run. Although the area still faces many obstacles, the field of optogenetics is growing rapidly and new advances are continuously being made to improve the technique (for a review see Dugue et al., 2012). For instance, optogenetic modulation in primate neurons has been investigated (Diester et al., 2011) and it has already been demonstrated that ChR2 can function within human neurons (Weick et al., 2010). Furthermore, results from optogenetic studies could be used to determine more effectively the specific region for applying Transcranial Magnetic Stimulation or Deep Brain Stimulation (DBS). Since in DBS all cells in a certain region are stimulated, many side effects have been reported (Frank et al., 2007; Serranová et al., 2013). Optogenetic stimulation would not result in such side effects because of its spatial specificity and could therefore potentially even replace DBS treatment in the future (Lalumiere, 2011). In addition, approaches like opto-fMRI might be translatable to humans in the medium term (Bullmore, 2012). Taken together, we believe that optogenetics complements other neuroscientific methods and should be used on a wider scale within cognitive neuroscience.
